# Age, environment, object recognition and morphological diversity of GFAP-immunolabeled astrocytes

**DOI:** 10.1186/s12993-016-0111-2

**Published:** 2016-10-10

**Authors:** Daniel Guerreiro Diniz, Marcus Augusto de Oliveira, Camila Mendes de Lima, César Augusto Raiol Fôro, Marcia Consentino Kronka Sosthenes, João Bento-Torres, Pedro Fernando da Costa Vasconcelos, Daniel Clive Anthony, Cristovam Wanderley Picanço Diniz

**Affiliations:** 1 Laboratório de Investigações Em Neurodegeneração e Infecção, Instituto de Ciências Biológicas, Universidade Federal do Pará, Hospital Universitário João de Barros Barreto, Rua dos Mundurucus 4487, Guamá, Belém, Pará CEP 66073-000 Brazil; 2 Departamento de Arbovirologia e Febres Hemorrágicas, Instituto Evandro Chagas, Ananindeua, Pará Brazil; 3 Laboratory of Experimental Neuropathology, Department of Pharmacology, University of Oxford, Oxford, England UK

**Keywords:** Environment, Exercise, Aging, Astrocytes morphology, Dentate gyrus, Memory

## Abstract

**Background:**

Few studies have explored the glial response to a standard environment and how the response may be associated with age-related cognitive decline in learning and memory. Here we investigated aging and environmental influences on hippocampal-dependent tasks and on the morphology of an unbiased selected population of astrocytes from the molecular layer of dentate gyrus, which is the main target of perforant pathway.

**Results:**

Six and twenty-month-old female, albino Swiss mice were housed, from weaning, in a standard or enriched environment, including running wheels for exercise and tested for object recognition and contextual memories. Young adult and aged subjects, independent of environment, were able to distinguish familiar from novel objects. All experimental groups, except aged mice from standard environment, distinguish stationary from displaced objects. Young adult but not aged mice, independent of environment, were able to distinguish older from recent objects. Only young mice from an enriched environment were able to distinguish novel from familiar contexts. Unbiased selected astrocytes from the molecular layer of the dentate gyrus were reconstructed in three-dimensions and classified using hierarchical cluster analysis of bimodal or multimodal morphological features. We found two morphological phenotypes of astrocytes and we designated type I the astrocytes that exhibited significantly higher values of morphological complexity as compared with type II. Complexity = [Sum of the terminal orders + Number of terminals] × [Total branch length/Number of primary branches]. On average, type I morphological complexity seems to be much more sensitive to age and environmental influences than that of type II. Indeed, aging and environmental impoverishment interact and reduce the morphological complexity of type I astrocytes at a point that they could not be distinguished anymore from type II.

**Conclusions:**

We suggest these two types of astrocytes may have different physiological roles and that the detrimental effects of aging on memory in mice from a standard environment may be associated with a reduction of astrocytes morphological diversity.

**Electronic supplementary material:**

The online version of this article (doi:10.1186/s12993-016-0111-2) contains supplementary material, which is available to authorized users.

## Background

Epidemiological studies have correlated physical and cognitive inactivity with a greater risk of age-related cognitive decline [1, 2]. In contrast, an active lifestyle may help prevent cognitive impairment in old age [3–5]; for recent reviews see [6–9]. Consistent with this view, the decline in memory that is associated with normal or pathological aging appears to be aggravated after institutionalization [10, 11]. Institutionalization is often associated with a standard-like environment with reduced sensory-motor and cognitive stimulation, social interactions, and physical activity, which contribute to a sedentary lifestyle [4, 5, 10, 12]. Similarly, it has been demonstrated that aged mice and rats, maintained in the standard environment of standard laboratory cages, perform worse in learning and memory tasks than those living in an enriched environment [13–24]. To perform spatial learning and memory tasks, the brain must accentuate the differences between old and new experiences, before coding occurs [25]. For that purpose, medial and lateral perforant pathways transmit to dentate gyrus, spatial and non-spatial information that would be necessary to recognize object placement (Where?), identity (What?) and timing (When?) [25].

Cellular and molecular analyses of these events demonstrate that the beneficial effects of environmental enrichment with voluntary exercise are associated with a variety of neuronal and neuroimmunological changes in both young and aged individuals [23, 26–35]. However, most of the documented changes in cell behavior relate to neuronal populations [36–40].

More recently significant contributions have explored possible roles of astrocytes in physiological and pathological brain aging [41]. Much of these outstanding work was done by Verkhratsky and Rodríguez-Arellano (see [104] for recent review), who showed that aging is associated with complex and region-dependent astrocyte remodeling, that may represent life-long adaptive responses [69] and that astrocytes participate in the morphological remodeling associated with synaptic plasticity [42]. However, astrocyte quantitative morphological studies under combined environmental and aging influences, in particular, is not yet largely explored [43–46].

A recent study showed that long-term potentiation and learning improved in chimeric mice generated by transplanting human astroglial progenitor cells into the forebrain [47]. In those chimeric mice, large regions of the CNS, including the hippocampus, consisted of mouse neurons (and oligodendrocytes) surrounded by human astrocytes and progenitor cells [48]. Both transplanted and control mice were then subject to a battery of learning and memory tasks and chimeric mice demonstrated enhanced performance on all tests. Those findings suggested that human astrocytes in particular, might contribute significantly, at least in part, to improved cognition [49, 50]. Morphologically, human astrocytes are larger and structurally more complex than mouse astrocytes [51]. Compared with mouse astrocytes, human astrocytes have soma diameters 2.6 mm longer, with tenfold more glial fibrillary acid protein (GFAP)-positive processes and fourfold faster calcium waves [52].

Taken together, these findings raise important questions related to the morphology of astrocytes and cognition. For example, is the performance of animals with more complexes immunolabeled astrocytes in the dentate gyrus, associated with better performances in object-identity tasks?

Thus, in the present report we described possible associations between environmental and age changes with alterations in the morphological complexity of GFAP immunolabeled astrocytes of the molecular layer of dentate gyrus, the main target of perforant pathway in mammals [53], and searched for potential associations between higher performances in the object recognition tests and higher morphological complexity of astrocytes.

## Methods

### Animals and experimental groups

More detailed experimental procedures have been previously described elsewhere [13]. Seventy-one Swiss female adult (6 months old—6 M) and aged (20 months old—20 M) mice were housed from 21st postnatal day either in enriched conditions (n = 42) or in standard conditions (n = 29). They remained as such until the sacrifice in each time window. These formed four experimental groups: enriched environment, young adults (EY, n = 12); standard environment, young adults (IY, n = 13); enriched environment, aged adults (EA, n = 30); and standard environment, aged adults (IA, n = 16). Enriched conditions comprised 2-level wire cages (100 × 50 × 100 cm) equipped with ropes, rod bridges, tunnels, running wheels, and toys. Toys were made of different forms of plastic, wood and metal of different colors, and were changed periodically. Each enriched cage housed 12-15 young and aged mice from housed from weaning in enriched conditions (EC, n = 27) or impoverished conditions (IC, n = 29). Water and food were delivered to the top and bottom levels, respectively. This obliged mouse to move from one compartment to another for drinking and eating. Standard conditions comprised plastic cages (32 × 39 × 100 cm) without equipment or toys. Each standard cage housed 12–13 young and aged mice. All mice had free access to water and food. In addition, 12-h dark and light cycles were maintained. Behavioral tests were administered during the light cycle.

### Object recognition tasks

#### Behavioral procedures

Current learning analyses do not use dynamic estimation methods and require many trials across many animals to assess significant differences in learning. Moreover, they provide no consensus on how best to identify when learning was occurred [54]. In the present work we used single trial tests to assess object recognition [55].

The apparatus for the single trial object recognition test consisted of an open box (30 × 30 × 40 cm) made of painted white wood. The floor was painted with lines to form nine squares (10 × 10 cm) and the luminance at the center of the cage floor was 2.4 cd/m^2^. Detailed protocols and reasons for test choices were discussed elsewhere [55, 56]; see also [57, 58] for reviews). In brief, behavioral essays were performed over 17 days: 7 days for handling, 3 days for open field habituation, 2 days for object habituation, and 5 days for testing: 1 day for each test. *Handling*: each day mice were placed in the center of the arena for 1 min and then removed to their cages. *Open field habituation*: each day mice were placed in the arena, free of objects, for 5 min to explore the open field. *Object habituation*: each day mice were exposed to two identical objects placed at the corners of the arena for 5 min, three times, with 50 min in between. These objects were not used on the test days. *Testing*: one-trial recognition tests were administered on five consecutive days: the object identity test, the object placement test, the object timing test, the context test, and the episodic-like memory test.

In order to minimize the influence of natural preferences for particular objects or materials, we chose objects of the same material but different geometries that could be easily discriminated and had similar possibilities for interaction [55]. All objects were plastic with different shapes, heights, and colors. Before each mouse entered the arena, the box and objects were cleaned with 75 % ethanol to minimize distinguishing olfactory cues.

*One*-*trial object identity recognition* consisted of a 5-min sample trial, during which subjects explored two identical objects in a familiar arena, followed by a 50-min intermission and then a second 5-min test trial, in which a “novel” object was presented together with one “familiar” object already explored during the sample trial. Objects differed in form, dimensions, color, and texture and had no ethological significance for mice.

*One*-*trial object placement recognition* followed the same procedure as above, except in the test trial, one of the two identical objects was shifted to a novel location (‘displaced” object).

*One*-*trial object timing recognition* consisted of three trials: two 5-min sample trials during which subjects explored two different object pairs; each trial was followed by a 50-min intermission; then one 5-min test trial in which one “former” and one “recent” object were presented together.

*One*-*trial object context recognition* also consisted of three trials: two 5-min sample trials and one 5-min test trial, each separated by 50 min intermissions. During the sample trials objects were presented in different ambient contexts. In the first sample trial two identical objects were presented under a bright light with extra-arena visual cues. In the second sample trial two different identical objects were presented under a dim light with different extra-arena visual cues. In the test trial two objects, one from each sample trial, were presented simultaneously in the bright light context [59].

All tests were video recorded by web cam and most images were analyzed with a computer program to score the time spent interacting with objects and the water maze performances (ANYMAZE tracking system, Stöelting). Computer analysis was done off-line. Exploration of an object was assumed when a mouse approached an object, the head was directed towards it, and the head was placed within 0–3 cm from the object. This definition required that each object be fixed to the apparatus floor, thus we chose heavy objects for interaction. Diagrams of the object recognition memory tests used in the present report are shown in Fig. 2 and the performance on each test is defined as the percentage of time spent exploring one object. To account for individual variability in exploratory activity, the time spent with each object was normalized by the total exploration time for each individual.

### Statistical analysis

Detailed statistical procedures for object recognition tests were described elsewhere [60]. Normality of the data distribution was tested and outliers were rarely removed from samples based on standard deviations. In brief, for object recognition tests, the basic measure obtained from video-images was the time a mouse spent exploring each object during the test trial, and scores were determined for recognition of identity (novel vs familiar), placement (displaced vs stationary), timing (recent vs former) and contextual (new context vs familiar context) memories. The data were analyzed by parametric statistics and the two-tailed t test for dependent groups was used to detect significant differences. The performance was the time of exploration for each object expressed as a proportion (percentage) of the total time of exploration, and possible significant differences were also detected with the two-tailed t test for dependent groups [59]. In all statistical tests the threshold for significance was set at p < 0.05.

### Perfusion and histological procedures

At the end of behavioral tests, 5–9 animals from each experimental group were weighed and killed with an overdose of ketamine (100 mg/kg) and xylazine (10 mg/kg) (Konig Laboratories). They were then perfused transcardially with heparinized saline for 10 min, followed by an aldehyde fixative (4 % paraformaldehyde in 0.1 M phosphate buffer, pH 7.2–7.4) for 30 min. All other chemicals were purchased from Sigma (São Paulo, Brazil). After perfusion and craniotomy, the brains were removed and cut on a vibratome (70 µm thickness). One of each five sections was used to detect GFAP by free-floating immunohistochemistry. Free-floating sections were rinsed once in 0.1 M phosphate buffer, transferred to 0.2 M boric acid pH 9.0, heated to 65–70 °C for 1 h, and then washed 3 × 5 min in 5 % PBST. The sections were incubated under constant gentle shaking in a 1 % hydrogen peroxide solution in methanol for 10 min, then rinsed 2 × 2 min in 0.1 M PBS. The sections were blocked with immunoglobulin for 1 h using the Mouse-on-Mouse Immunodetection kit (M.O.M. kit, Vector Laboratories, USA) according to the manufacturer’s instructions. Blocking was followed by washing for 3 × 2 min in PBS. Sections were incubated in a working solution of protein concentrate for 5 min, then incubated with monoclonal mouse anti-GFAP primary antibody (MAB360, CHEMICON Int., USA), diluted in protein concentrate solution (M.O.M. kit), at 4 °C for 3 days with continuous, gentle agitation. Next, the sections were washed 3 × 2 min in PBS and incubated for 20 h with biotinylated horse anti-mouse secondary antibody (M.O.M. kit), diluted 1:100 in PBS. After washing 3 × 2 min in PBS, sections were transferred to an avidin–biotin-peroxidase complex solution (ABC, Vector Laboratories, USA, 1:200) for 1.5 h, washed 3 × 2 min in 0.1 M PBS, and processed with the glucose oxidase-DAB-nickel method and peroxidase histochemistry [61].

The reaction was interrupted after fine astrocytic branches were detected under the microscope. Sections were rinsed 4 × 5 min in 0.1 M PBS, mounted on gelatinized slides, dehydrated in alcohol and xylene, and coverslipped with Enthelan (Merck). Five animals from each group with complete GFAP immunohistochemistry slide collections that contained conspicuous morphological details of astrocytes were used for 3-D reconstruction and morphometric analysis.

### 3-D astrocyte reconstruction and quantitative morphology

We selected five brains from each experimental group for GFAP immunolabeling and 3-D reconstruction. To analyze brain sections, we used a NIKON Eclipse 80i microscope (Nikon, Japan) equipped with a motorized stage (MAC6000, Ludl Electronic Products, Hawthorne, NY, USA). Astrocytes from the layer of interest were analyzed under oil immersion, with a high-resolution, 100 × oil immersion, plan fluoride objective (Nikon, NA 1.3, DF = 0.19 µm). Images were acquired with Neurolucida and analyzed with Neurolucida explorer software (MBF Bioscience Inc., Frederick, MD, USA). Although shrinkage in the z-axis is not a linear event, we corrected the shrinkage in the z-axis, based on previous evidence of 75 % shrinkage [62]. Without correction, this shrinkage would significantly distort the length measurements along this axis. Only cells with processes that were unequivocally complete were included for 3-D analysis; cells were discarded when branches appeared artificially cut or not fully immunolabeled. Terminal branches were typically thin.

### Morphometric analysis and statistics

**Fig. 1:**
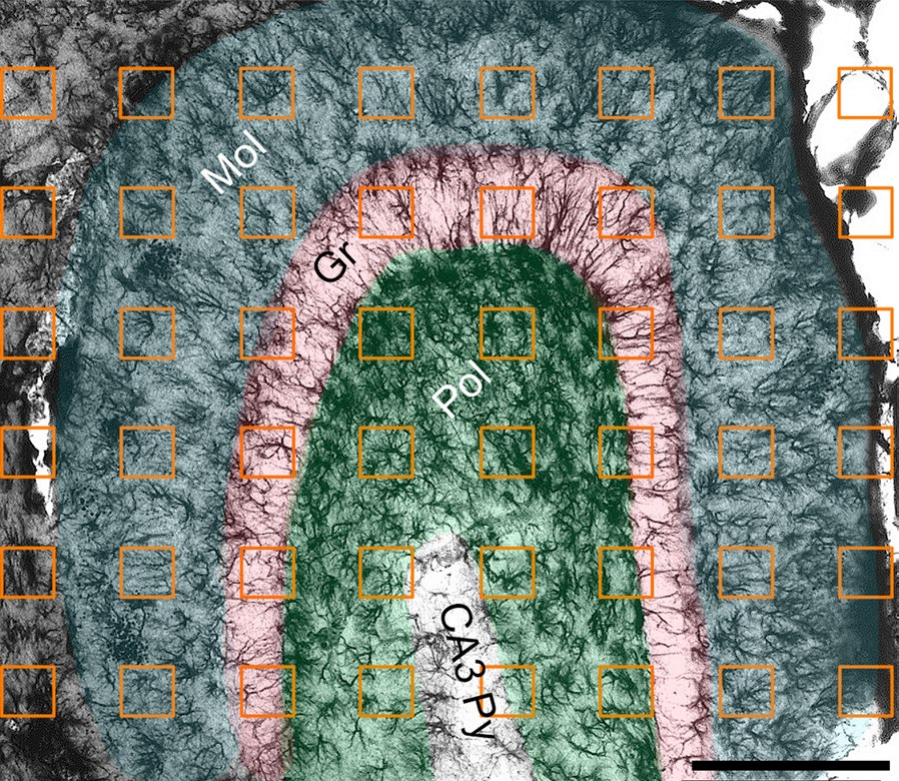
Low-power photomicrograph of mouse dentate gyrus from a section immunolabeled with anti-GFAP antibody to reveal the laminar distribution of astrocytes and to define the layers and limits of the dentate gyrus. Note the boundaries of the granular layer (Gr, *pink*) are demarcated by adjacent molecular (Mol, *blue*) and polymorphic (Pol, *green*) layers. Reduced GFAP immunostaining in the CA3 pyramidal layer (CA3Py) clearly delineates the boundary between the polymorphic layer and the pyramidal layer. The grid (straight *green lines* parallel to the *x-* and *y-axes*) establishes the intervals between the* orange square boxes* and illustrates the random and systematic sampling approach. The* number of boxes* in each section is proportional to the area covered by the dentate gyrus. A single astrocyte located inside every* box* was selected for three-dimensional reconstruction. *Scale bar* 250 μm

To accomplish the analysis, we used 20 animals, five from each experimental group (IY, n = 5; EY, n = 5; EA, n = 5; IA, n = 5). From these four groups, we digitally reconstructed 309 astrocytes in three-dimensions from the molecular layer (EY = 79, IY = 75, EA = 76, IA = 79) of the dentate gyrus. Astrocytes for 3-D reconstructions were selected in an unbiased, randomized, and systematic way (Fig. 1). We used architectonic differences in the neuropil region, readily visible in immunolabeled sections, to define the limits of the dentate gyrus layers of the hippocampus. Systematic and random samples were taken from a series of sections containing dorsal and ventral dentate gyrus to guarantee that all regions had the same probability of being included among the analyzed samples. Each box inside the outlined dentate gyrus layers indicates a site from which we selected a single astrocyte for 3-D reconstruction (Fig. 1).

We first investigated the presence of morphological features shared by the astrocytes observed in each layer of interest in our sample, inside each experimental group. We selected all morphometric quantitative variables with multimodality indices (MMI) higher than 0.55, to an initial cluster analysis (Ward’s hierarchical clustering method), which included all animals from each group. To estimate the multimodality index (MI) based on skewness and kurtosis of our sample for each morphometric variable as previously defined elsewhere: MI = [M3^2^ + 1]/[M4 + 3 (n − 1)^2^/(n − 2) (n − 3)], where M3 is skewness and M4 is kurtosis and n is sample size [63, 64]. Kurtosis and skewness describe the shape of the data distribution and enable to distinguish between unimodal, bimodal or multimodal curves. Multimodal data sets are essential for separating a population of cells into cell types [63]. The multimodal index of each variable was estimate based on the measurements of 30 morphometric features of astrocytes, 10 related to the soma and 20 to the branches, as follows: 1. Soma area (µm^2^); 2. Soma perimeter; 3. Feret minimum diameter; 4. Feret Mouse maximum diameter (maximum diameter in a shape); 5. Compactness; 6. Form factor; 7. Solidity; 8. Roundness; 9. Aspect ratio; 10. Convexity; 11. Branch length (µm); 12. Total tree length (µm) 13. Surface area (µm^2^); 14. Branch volume (µm^3^); 15. Segments/mm; 16. Tortuosity; 17. Fractal dimensions (k-dim); 18. Base diameter of the primary branch (µm); 19. Total number of segments; 20. Number of varicosities; 21. Planar angle; 22. Number of trees; 23. Complexity; 24. Convex hull volume; 25. Convex hull surface; 26. Convex hull area; 27. Convex hull perimeter; 28. Vertex Va; 29. Vertex Vb; 30. Vertex Vc. Table 1 contains descriptions of all morphometric variables used.

**Table 1:** Morphometric features definitions

*Branched structure analysis*	
Segment	Any portion of microglial branched structure with endings that are either nodes or terminations with no intermediate nodes
Segments/mm	Number of segments/total length of the segments expressed in millimeters
No of trees	Number of trees in the astrocytes
Total no of segments	Refer to the total number of segments in the tree
Branch length	Total length of the line segments used to trace the branch of interest.
Total branch length	Total length for all branches in the tree Mean = [length]/[number of branches]
Tortuosity	=[Actual length of the segment]/[distance between the endpoints of the segment]. The smallest value is 1; this represents a straight segment. Tortuosity allows segments of different lengths to be compared in terms of the complexity of the paths they take
Surface area	Computed by modeling each branch as a frustum (truncated right circular cone)
Tree surface area	
Branch volume	Computed by modeling each piece of each branch as a frustum.
Total branch volume	Total volume for all branches in the tree
Base diameter of primary branch	Diameter at the start of the 1st segment
Planar Angle	Computed based on the endpoints of the segments. It refers to the change in direction of a segment relative to the previous segment
Fractal dimension	The “k-dim” of the fractal analysis, describes how the structure of interest fills space. Significant statistical differences in k-dim suggest morphological dissimilarities
Convex hull-perimeter	Convex hull measures the size of the branching field by interpreting a branched structure as a solid object controlling a given amount of physical space. The amount of physical space is defined in terms of convex-hull volume, surface area, area, and or perimeter
Vertex analysis	Describes the overall structure of a branched object based on topological and metrical properties. Root (or origin) point: For neurons, microglia or astrocytes, the origin is the point at which the structure is attached to the soma. Main types of vertices: V_d_ (bifurcation) or V_t_ (trifurcation): Nodal (or branching) points. V_p_: Terminal (or pendant) vertices. V_a_: primary vertices connecting 2 pendant vertices; V_b_: secondary vertices connecting 1 pendant vertex (V_p_) to 1 bifurcation (V_d_) or 1 trifurcation (V_t_); V_c_: tertiary vertices connecting either 2 bifurcations (V_d_), 2 trifurcations (V_t_), or 1 bifurcation (V_d_) and 1 trifurcation (V_t_). In the present report we measure the number of vertices Va, Vb and Vc
Complexity	Complexity = [sum of the terminal orders + number of terminals] × [total branch length/number of primary branches]
*Cell body*	
Area	Refers to the 2-dimensional cross-sectional area contained within the boundary of the cell body
Perimeter	Length of the contour representing the cell body
Feret max/min	Largest and smallest dimensions of the cell body as if a caliper was used to measure across the contour. The two measurements are independent of one another and not necessarily at right angles to each other
Aspect ratio	Aspect ratio = [min diameter]/[max diameter] Indicates the degree of flatness of the cell body Range of values is 0–1 A circle has an aspect ratio of 1
Compactness	Compactness = $\frac{{\sqrt {\left( {\frac{4}{\pi }} \right)} \times Area}}{Max Diam}$ The range of values is 0–1 A circle is the most compact shape (compactness = 1)
Convexity	Convexity = [convex perimeter]/[perimeter] A completely convex object does not have indentations, and has a convexity value of 1 (e.g., circles, ellipses, and squares) Concave objects have convexity values less than 1 Contours with low convexity have a large boundary between inside and outside areas
Form factor	$Form factor = 4\pi \times \frac{Area}{{perimeter^{2} }}$ As the contour shape approaches that of a perfect circle, this value approaches a maximum of 1.0 As the contour shape flattens out, this value approaches 0
Roundness	Roundness = [compactness]^2^ Use to differentiate objects that have small compactness values
Solidity	Solidity = [area]/[convex Area] The area enclosed by a ‘rubber band’ stretched around a contour is called the convex area Circles, squares, and ellipses have a solidity of 1 Indentations in the contour take area away from the convex area, decreasing the actual area within the contour

We found that a few microglial morphological features showed a multimodality index greater than 0.55 and this index value indicates that the distribution is at least bimodal and may be multimodal, and these particular features were selected for cluster analysis as previously described [63]. We used the Ward’s method with standardized variables, square Euclidian distances and a tree diagram (dendrogram) to illustrate the classification generated by cluster analysis. From hierarchical cluster analysis we categorized astrocytes into two groups designated types I and II.

We applied this multivariate statistical procedure to our sample of astrocytes in order to search for potential astroglial morphological classes inside of each experimental group. The classification of astrocytes suggested by cluster analysis was assessed using a forward stepwise discriminant function analysis performed with Statistica 12.0 (Statsoft, Tulsa, OK). Discriminant function analysis was used to determine which variables discriminate between two or more naturally occurring groups. The purpose of this procedure is to determine whether the groups differ with regard to the mean of a variable, and then to use that variable to predict group membership. In the present study, we used this software to perform comparisons between matrices of total variances and co-variances. These matrices were compared using multivariate F tests to determine whether there were any significant between-group differences (with regard to all variables). In the step-forward discriminant function analysis, the program builds a model of discrimination step-by-step. In this model, at each step, all variables are reviewed and evaluated to determine which variable contributes most to the discrimination between groups. We applied this procedure to determine morphometric variables that provided the best separation between the astroglial classes suggested by the cluster analysis. In addition, we calculated the arithmetic mean and standard deviation for the variables chosen as the best predictors for the astroglial groups. Parametric statistical analyses with t tests were applied to compare groups of astrocytes inside each experimental group and to detect possible morphological differences between average astrocytes from molecular layer of each experimental group. In the selected sections, the margins of the polymorphic, granular, and molecular layers were clearly distinguished with Nissl counterstaining.

All astrocytes from each layer of interest were measured multiple times, and dedicated software (Neurolucida explorer, MicroBright Field Inc.) was used to process data obtained with Neurolucida.

## Results

### Behavioral outcomes

**Fig. 2:**
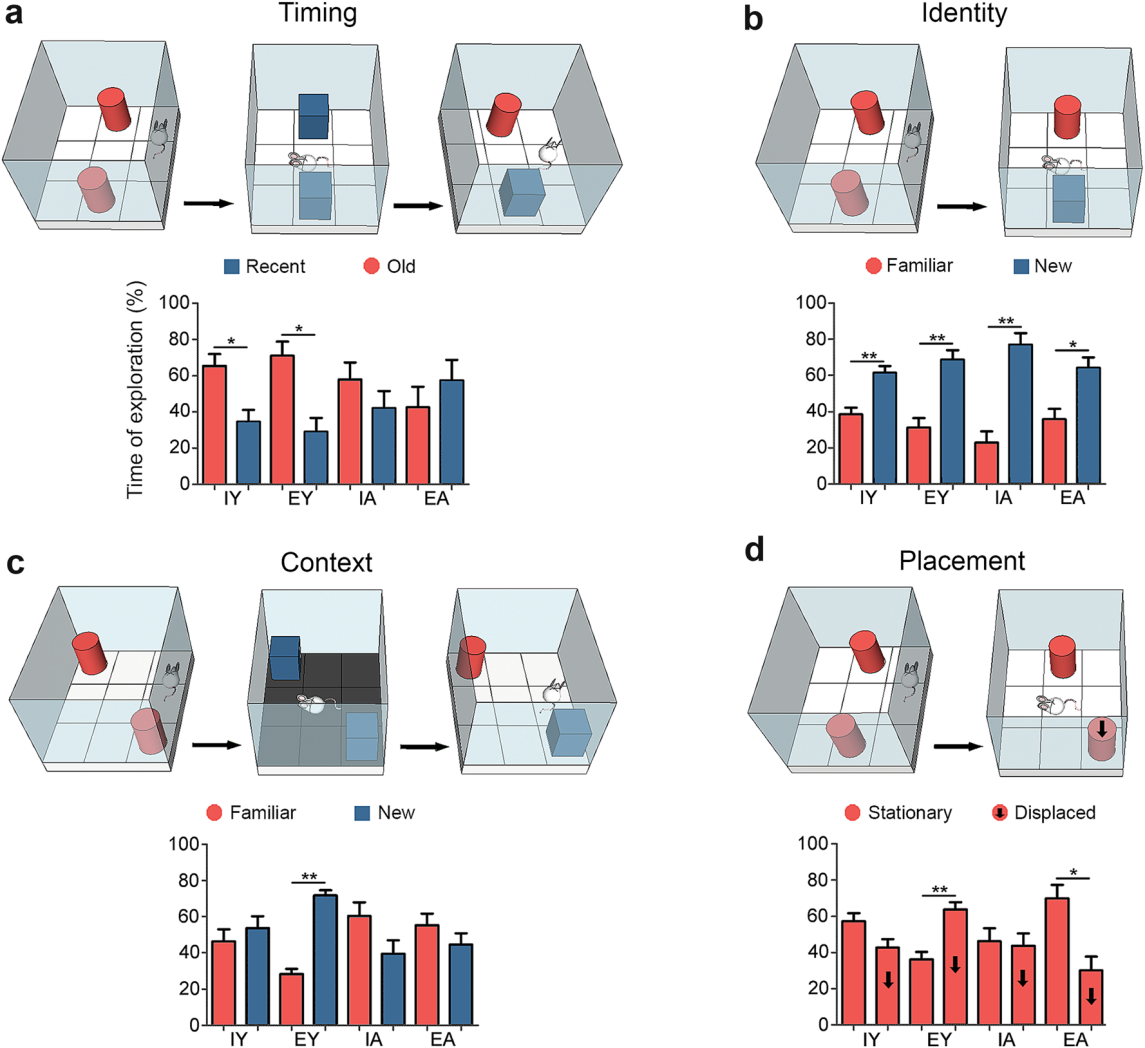
Object recognition and contextual memories. **a** Timing; **b** Identity; **c** Context; **d** Placement. *Bars* indicate average values of the exploration time ± s.e. for each group. *Red* and *blue filled bars* represent differences between objects (displaced vs. stationary, old vs. recent, familiar vs. new). Two-tailed t test for dependent samples; *p < 0.05; **p < 0.001. *A SE* aged mice from standard environment; *A EE* aged mice from enriched environment; *Y SE* young mice from standard environment; *Y EE* young mice from enriched environment

Results of the object recognition tests are shown in Fig. 2. All experimental groups were able to distinguish familiar from new objects (identity—What?). All other tasks were significantly influenced by age, environment, or both as follow:

#### One trial object timing recognition

Young adult mice, independent of environment, were able to distinguish older from recent objects (When?) (Y SE: t = 2.38, p = 0.0411; Y EE: t = 2.72, p = 0.0235). In contrast, aged mice independent of environment were unable to make this distinction (A SE: t = 0.83, p = 0.426; A EE: t = 0.66, p = 0.526).

#### One-trial object identity recognition

Young adult and aged subjects, independent of environment, were able to distinguish familiar from novel objects. Y SE: t = 4.49, p = 0.0015; Y EE: t = 3.60, p = 0.0058; A SE: t = 4.30, p = 0.0020; A EE: t = 2.45, p = 0.0364.

#### One trial object context recognition

Only young mice from an enriched environment were able to distinguish novel from familiar contexts. Y SE: t = 0.033, p = 0.973; Y EE: t = 7.56, p < 0.0001; A SE: t = 1.39, p = 0.201; A EE: t = 0.87, p = 0.406.

#### One trial object placement recognition

Aged mice from standard conditions were unable to distinguish stationary from displaced objects (A SE: t = 0.274, p = 0.789), however young mice, raised in similar conditions were able to do so (Y SE: t = 2258, p = 0.0503). In contrast, animals from enriched environments, independent of age, were able to distinguish stationary from displaced objects (Where?) with different preferences: young mice spent more time with displaced objects and aged mice with stationary objects (Y EE: t = 3.38, p = 0.0081; A EE: t = 2.62, p = 0.0305). Additional file 1: Table S1 shows absolute values of time of exploration on each hippocampal-dependent task for all experimental groups.

### Morphological phenotypes of astrocytes in the molecular layer of dentate gyrus

**Fig. 3:**
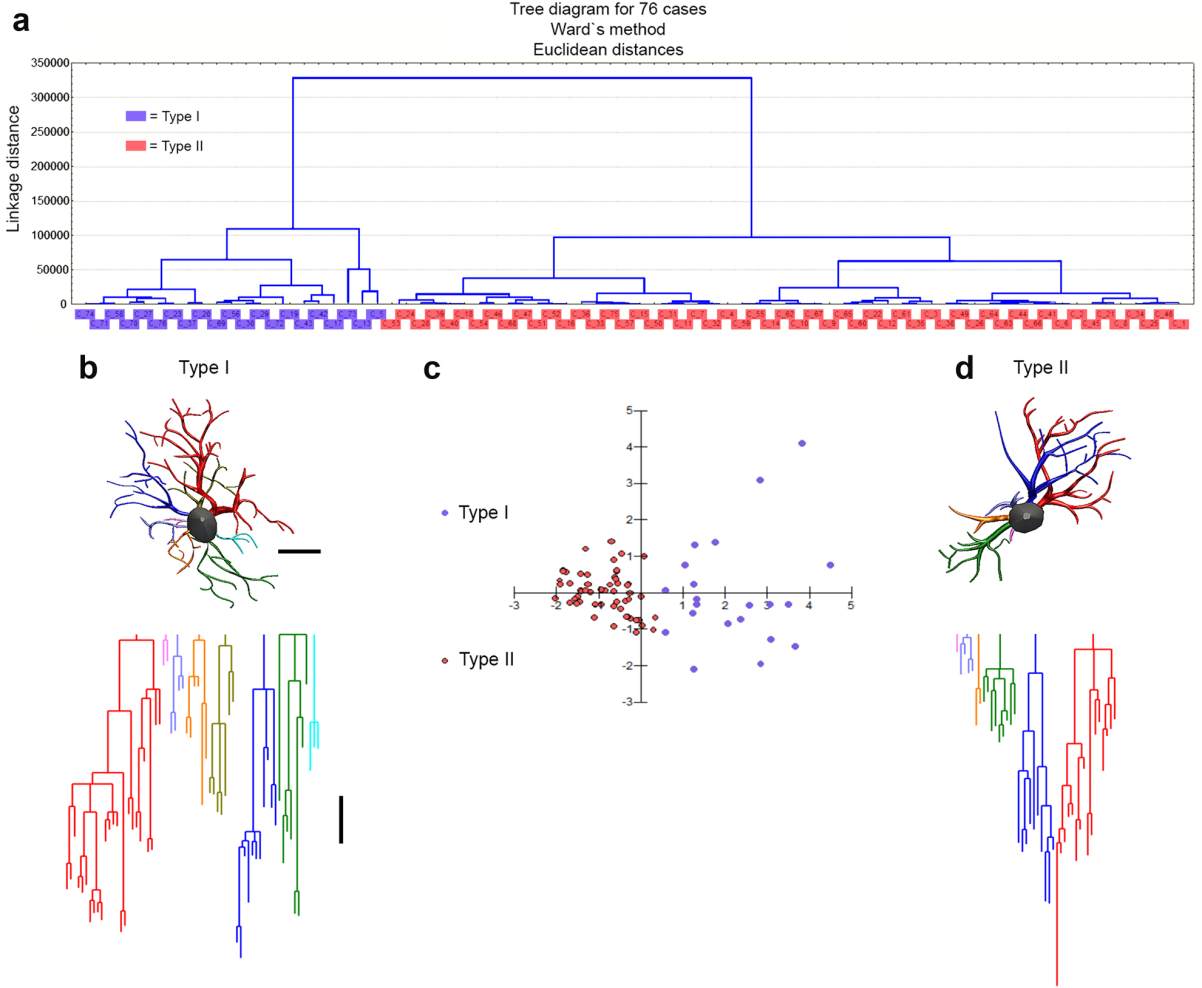
Morphological phenotypes of astrocytes in the molecular layer of the dentate gyrus (MolDG) of 6 mo. adult mice raised in an enriched environment (Y EE mice). Cluster discriminant analysis (Ward’s method) and three-dimensional reconstructions of MolDG astrocytes from five Y EE mice. **a** Dendrogram groupings of 76 dentate gyrus astrocytes indicated two main morphological phenotypes (type I and type II). **b** Three-dimensional reconstruction of an astrocyte with mean values closer to the mean values of morphometrical features of type I astrocyte. **c** Graphic representation of the discriminant analysis. The variables that contributed most to cluster formation were complexity (1 × 10^−9^) and convex-hull volume (p < 0.00001). Type I (*blue dots*) showed higher X–Y dispersion than Type II (*orange dots*) astrocytes. Astrocytes were reconstructed from both rostral and caudal regions of the dentate gyrus; cluster analysis was based on multimodal or at least bi-modal morphometric features of astrocytes (MMI >0.55). **d** Three-dimensional reconstruction of an astrocyte with mean values closer to the mean values of morphological features of type II astrocyte. Below the three-dimensional reconstructions are the corresponding linear dendrograms of each arbor of astrocytes type I and II. The length of each branch segment is displayed to scale as vertical lines; sister branches are horizontally displaced. The dendrogram was plotted and analyzed using Neuroexplorer (MicroBrightField). Branches of the same parental (primary branch) trunk are shown in *one color*. Note that the type I astrocyte is more complex than the type II astrocyte. *Y EE* young mice from enriched environment. *Scale bars* 10 μm

**Fig. 4:**
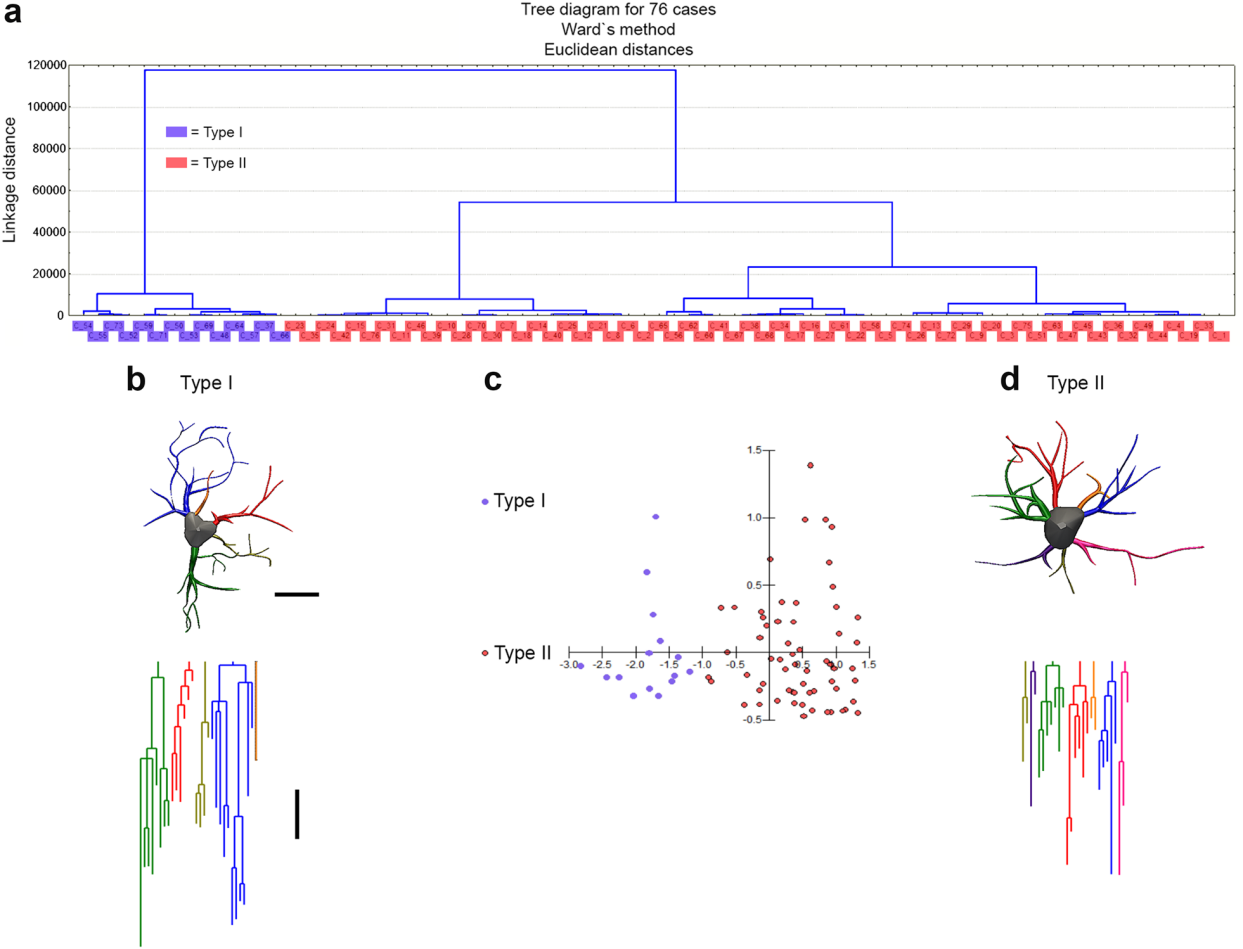
Morphological phenotypes of astrocytes in the molecular layer of the dentate gyrus (MolDG) of 6 mo. adult mice raised in standard environment (Y SE mice). Cluster discriminant analysis (Ward’s method) and three-dimensional reconstructions of MolDG astrocytes from five Y SE mice. **a** Dendrogram groupings of 76 dentate gyrus astrocytes indicated two main morphological phenotypes (type I and type II). **b** Three-dimensional reconstruction of an astrocyte with mean values closer to the mean values of morphometrical features of type I astrocyte. **c** Graphic representation of the discriminant analysis. The variable that contributed most to cluster formation was convex-hull volume (p < 0.016). Type I (*blue dots*) showed similar X–Y dispersion as compared with Type II (*orange dots*) astrocytes. Astrocytes were reconstructed from both rostral and caudal regions of the dentate gyrus; cluster analysis was based on multimodal or at least bi-modal morphometric features of astrocytes (MMI >0.55). **d** Three-dimensional reconstruction of an astrocyte with mean values closer to the mean values of morphological features of type II astrocyte. Below the three-dimensional reconstructions are the corresponding linear dendrograms of each arbor of astrocytes type I and II. The length of each branch segment is displayed to scale as vertical lines; sister branches are horizontally displaced. The dendrogram was plotted and analyzed using Neuroexplorer (MicroBrightField). Branches of the same parental (primary branch) trunk are shown in *one color*. Note that the type I astrocyte is more complex than the type II astrocyte. *Y SE* young mice from standard environment. *Scale bars* 10 μm

**Fig. 5:**
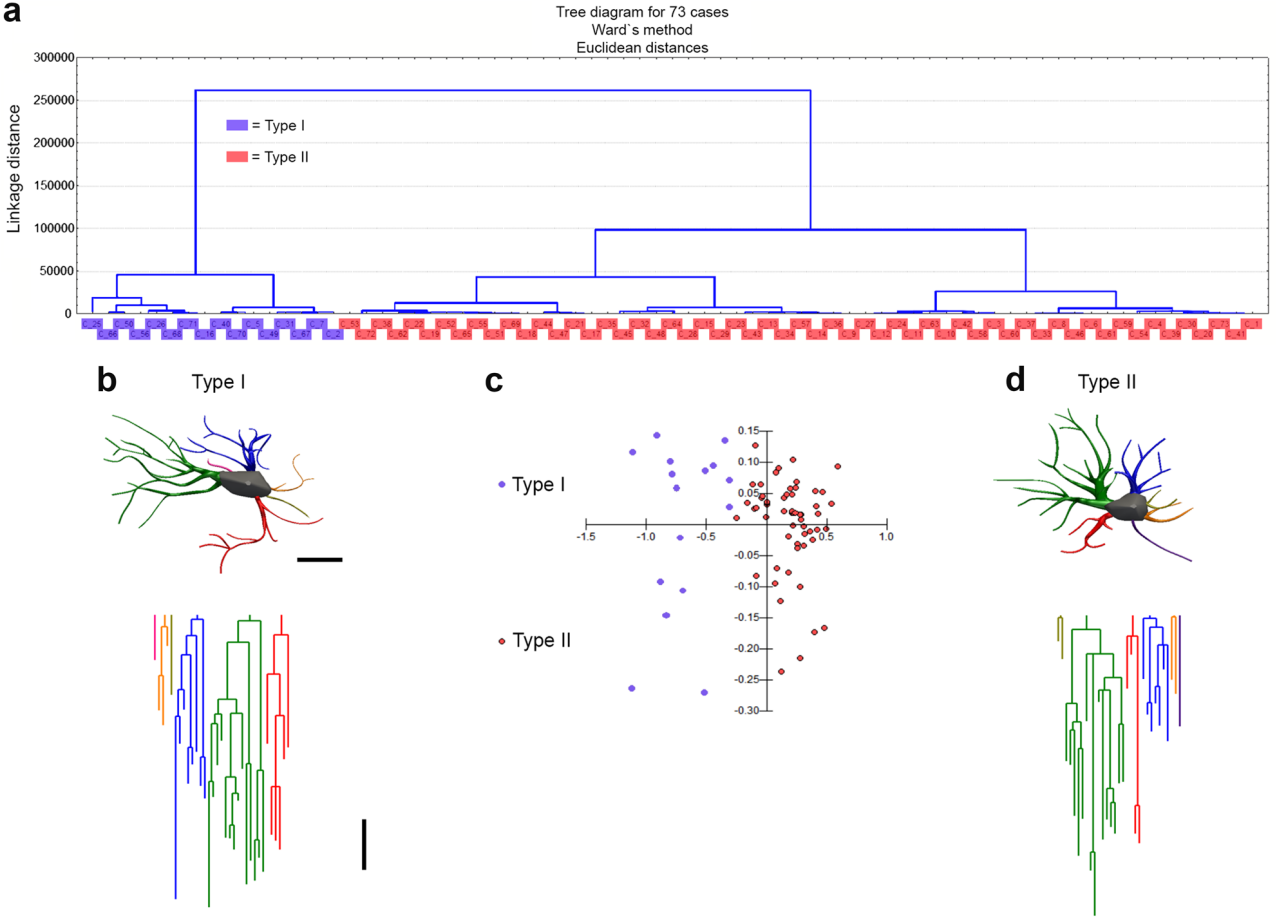
Morphological phenotypes of astrocytes in the molecular layer of the dentate gyrus (MolDG) of aged mice raised in enriched environment (A EE mice). Cluster discriminant analysis (Ward’s method) and three-dimensional reconstructions of MolDG astrocytes from five A EE mice. **a** Dendrogram groupings of 73 dentate gyrus astrocytes indicated two main morphological phenotypes (type I and type II). **b** Three-dimensional reconstruction of an astrocyte with mean values closer to the mean values of morphometrical features of type I astrocyte. **c** Graphic representation of the discriminant analysis. The variable that contributed most to cluster formation was complexity (p < 0.46 × 10^−26^). Type I (*blue dots*) showed higher X–Y dispersion than Type II (*orange dots*) astrocytes. Astrocytes were reconstructed from both rostral and caudal regions of the dentate gyrus; cluster analysis was based on multimodal or at least bi-modal morphometric features of astrocytes (MMI >0.55). **d** Three-dimensional reconstruction of an astrocyte with mean values closer to the mean values of morphometrical features of type II astrocyte. Below the three-dimensional reconstructions are the corresponding linear dendrograms of each arbor of astrocytes type I and II. The length of each branch segment is displayed to scale as vertical lines; sister branches are horizontally displaced. The dendrogram was plotted and analyzed using Neuroexplorer (MicroBrightField). Branches of the same parental (primary branch) trunk are shown in *one color*. *A EE* aged mice from enriched environment. *Scale bars* 10 μm

**Fig. 6:**
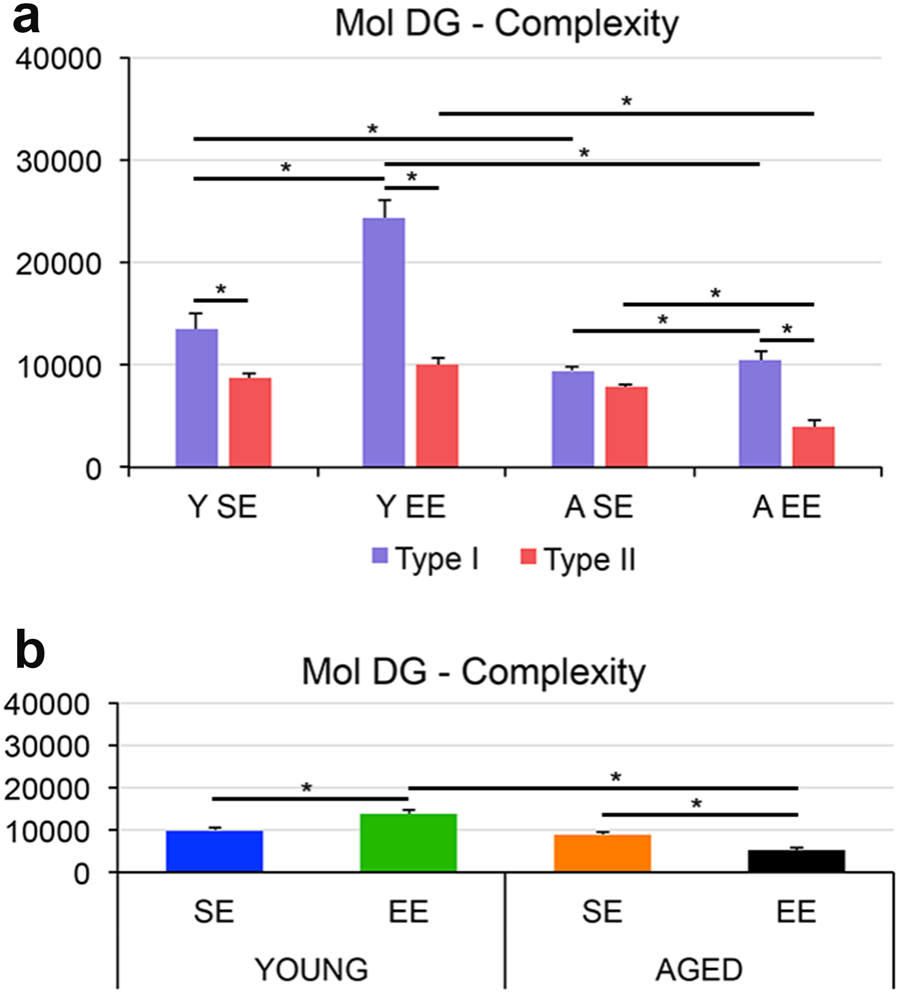
Environment and aging influences on the morphology of astrocytes from the dentate gyrus. **a** Graphic representations of mean and standard error values of morphological complexity of the astrocytes Types I and II from molecular layer (Mol DG) of dentate gyrus. Significant differences between appropriate comparisons are indicated by *bar connections* with an *asterisk*. **b** Graphic representation of mean and standard errors values of complexity of the “mean” astrocyte of each experimental group. *Y SE* young adult raised in standard environment; *Y EE* young adult raised in enriched environment; *A SE* aged mice raised in standard environment and *A EE* aged mice raised in enriched environment. *SE* standard environment; *EE* enriched environment

We used microscopic 3-D reconstructions and an unbiased, systematic, randomized sampling approach to select astrocytes from the molecular layers of the dentate gyrus. Cluster and discriminant analysis illustrate these findings, together with 3-D reconstructions of astrocytes with morphological features close to the “mean astrocyte” of each experimental group. These findings are shown in Figs. 3, 4, 5, 6.

Based on morphometric features and hierarchical cluster analysis using multimodal parameters, we proposed to designate type I and type II as a function of their morphological complexities. As compared to type II, type I was the group of astrocytes with significant higher mean values of complexity. Table 2 summarizes discriminant analysis results and reveals that a few morphological measurements are enough to distinguish Type I from Type II astrocytes in the molecular layer of different experimental groups. Among them, morphological complexity was the morphological feature that contributed most to cluster formation. Complexity is a combination of different morphological features of astrocytes trees. Longer and more ramified astrocytes show higher values of complexity. Based on this parameter we measured the influence of aging and environment on dentate gyrus astrocytes morphology.

**Table 2:** Discriminant analysis summary to indicate the morphological variables that most contribute to cluster formation of types I and II astrocytes from the molecular layer of dentate gyrus of each experimental group

	Wilks’	Partial	F-remove	p level	Toler.	1-Toler.
*Molecular layer*						
Y EE						
Complexity	0.449649	0.574029	53.429	0.0000000003	0.809881	0.19012
Convex hull volume (µm^3^)	0.337713	0.764293	22.205	0.0000116304	0.826130	0.17387
Y SE						
Convex hull volume (µm^3^)	0.550693	0.4469105	84.15575	0.00000000000016	0.952663	0.04734
A EE						
Complexity	0.9949113	0.2732307	194.1735	2.946224E^−22^	0.9189208	0.08108

Because astrocytes from the molecular layer of aged mice from standard environment (A SE) were morphologically quite homogeneous (very short Euclidian distances), data is not included here

*Y EE* young mice from enriched environment; *Y SE* young mice from standard environment; *A EE* aged mice from enriched environment

Figure 3a represents the hierarchical cluster analysis of the molecular layer astrocytes morphological features from young mice raised in enriched environment. As previously mentioned, this analysis was done using the morphological parameters with MMI >0.55 as follow: branch volume, aspect ratio, convexity, form factor, complexity, convex hull volume, convex hull surface, convex hull area. Two main clusters of astrocytes were distinguished in the molecular layer of this group and the variables that most contributed to their formation were complexity (p < 0.29 × 10^−9^) and convex hull volume (p < 0.12 × 10^−4^). The astrocyte features corresponding to clusters I (Fig. 3b) and II (Fig. 3D) where the presence of significant differences in complexity and convex hull volumes. Figure 3c is a graphic representation of the discriminant analysis to illustrate the distribution of astrocytes in X–Y plot. Note that type I astrocytes dots are more dispersed than type II and the spatial distribution of type I and II dots are quite distinct. Similar analysis was applied to the astrocytes from young adult raised in standard cages (Fig. 4a–d), and to the astrocytes from aged mice raised in enriched (Fig. 5a–d) or in standard cages (not illustrated). Except for the aged mice group from standard environment, showing a single morphological phenotype, all other cases showed two distinct astrocyte morphologies, with notable differences in the mean values of complexity. Indeed, because molecular layer astrocytes from aged mice raised in standard environment were morphologically homogeneous with small Euclidian distances, we could not distinguish type I from type II astrocytes in this group (not illustrated). Surprisingly, in relative terms, the reduction of morphological complexity in both type I and II astrocytes was higher in aged mice maintained in enriched environment than in aged mice from standard environment (Fig. 6). We found no difference between type I and II astrocytes in the molecular layer of aged mice from a standard environment and this was associated with spatial memory impaired performance. In contrast, we still detected significant differences between type I and II astrocytes in aged animals from enriched environment and this was associated with intact spatial memory suggesting that the morphological diversity of astrocytes may be important to maintain spatial memory integrity.

### Influences of environment and age on the morphological complexity of astrocytes in the dentate gyrus

Complexity has been defined previously [65] using the following equation:

$$\begin{aligned} {\text{Complexity}} = \left[ {{\text{Sum of the terminal orders}} + {\text{Number of terminals}}} \right] \hfill \\ \;\;\;\;\;\;\;\;\;\;\;\;\;\;\;\;\;\;\; \times \left[ {{\text{Total branch length}}/{\text{Number of primary branches}}} \right]. \hfill \\ \end{aligned}$$

See http://www.mbfbioscience.com/help/nx11/Default.htm#Analyses/BranchedStructure/neuronSumm.htm for details.

As previously mentioned, more ramified and longer astrocytes are given higher values of complexity. Based on cluster and discriminant analysis we categorized astrocytes into two groups with respect to complexity, and designated as type I the astrocytes that exhibited significantly higher values of complexity in comparison with type II.

Figure 6 and Tables 3 and 4 demonstrate the influences of age and environmental effects on the complexity of type I and II astrocytes (A–C) and on the “mean astrocyte’ (D–F). In the last case complexity represents the mean of complexity of all astrocytes (without distinction between type I and II). Two-way ANOVA applied to the “mean astrocyte’ complexity values revealed that aging and environmental impoverishment, acting together, reduces astrocytes complexity (Table 3). However, the analysis of aging and environment influences on complexity of type I and II astrocytes separately (Fig. 6), revealed that the long term effects of aging and environment seems to affect type I and type II astrocytes from aged mice raised in enriched environment to a greater extent than astrocytes from aged mice maintained in standard environment (Table 4). Indeed, in the molecular layer, except for the A-SE group, which was submitted to impoverishment environment throughout life, type I cells were preserved quite distinct from type II in terms of complexity in all groups. Type I astrocytes were more complex in young adults than in aged groups and more complex in aged mice raised in enriched environment than in standard environment. However, type I astrocytes complexity mean values were not different from type II values in aged mice raised in standard environment.

**Table 3:** Influences of age and environment on the morphological complexity of the “mean astrocyte” from molecular layer of dentate gyrus

Molecular layer of dentate gyrus	F	P		
Age	49.529	0.000		
Environment	0.091	0.763		
Age and environment	32.231	0.000		
Two-tail t test	*Y EE x Y SE*	*Y EE x A EE*	*Y SE x A SE*	*A EE x A SE*
t=	34,475	85,256	10,210	−53,432
p=	0.001	<0.0001	0.309	<0.0001

Two-way ANOVA with correspondent F and p values and two-tail t tests with correspondent t and p values

*Y EE* young mice from enriched environment; *Y SE* young mice from standard environment; *A EE* aged mice from enriched environment; *A SE* aged mice from standard environment

**Table 4:** Influences of age and environment on the morphological complexity of Type I and Type II astrocytes from molecular layer of dentate gyrus (Mol-DG)

Mol-DG type I × II	Y SE	Y EE	A SE	A EE
t=	30,689	82,001	−14,087	129,240
p=	0.003	<0.0001	0.1629	<0.0001
Type I	*Y EE X Y SE*	*Y EE X A EE*	*Y SE X A SE*	*A EE X A SE*
t=	45,419	82,187	32,042	27,412
p=	<0.0001	<0.0001	0.003	0.010
Type II	*Y EE X Y SE*	*Y EE X A EE*	*Y SE X A SE*	*A EE X A SE*
t=	15,521	101,537	−0.8054	−70,682
p=	0.1234	<0.0001	0.4224	<0.0001

Two-tail t tests with correspondent t and p values

*Y EE* young mice from enriched environment; *Y SE* young mice from standard environment; *A EE* aged mice from enriched environment; *A SE* aged mice from standard environment

**Fig. 7:**
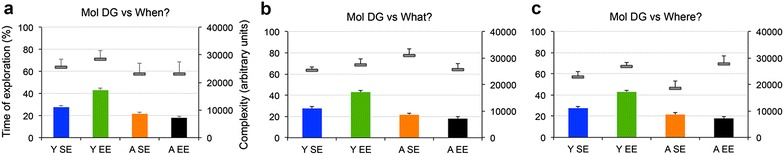
Object identity recognition (What?), timing (When?), spatial memory (Where?), and astrocytes complexity. Object discrimination index is expressed as percentage values on the left *Y-axis* and astrocyte morphological complexity is indicated as arbitrary values on the right *Y-axis*. Discrimination index of 60 % or higher was set to indicate that mice distinguished between the objects (familiar vs new; stationary vs displaced; old vs recent) whereas indices below 60 % indicate no object recognition. *Mol DG* molecular layer of dentate gyrus, *Y SE* young mice from standard environment, *Y EE* young mice from enriched environment, *A SE* aged mice from standard environment, *A EE* aged mice from enriched environment. **a** Object timing recognition and astrocytes morphological complexity. **b** Object identity recognition and astrocytes morphological complexity. **c** Object placement recognition and astrocytes morphological complexity

No simple correlations were detected between identity, placement or timing test results and the morphological complexity (Fig. 7). Similar analysis applied separately to type I or II astrocytes complexities and behavioral performances (not illustrated) showed similar negative results suggesting that cognition and morphological complexity of dentate gyrus astrocytes may not be linearly related.

## Discussion

We used stereological random and systematic sampling [66], combined with 3-D reconstruction of astrocytes to show that astrocyte morphological complexity is experience-dependent. We also demonstrated that young mice with more complex astrocyte structures showed, on average, better performance in object recognition tests. To our knowledge, there are no previous findings that result from applying an unbiased sample approach with 3-D microscopic reconstruction to the assessment of an astrocytes’ morphological phenotype in the mouse dentate gyrus. This approach was chosen to guarantee that all regions from the area of interest would have the same probability of inclusion in the (systematic and randomized) sample, and that fine anatomical details (from 3-D reconstructed astrocytes) could be quantified in all experimental groups using unbiased methods. From this sampling approach, associated with cluster and discriminant analysis of the morphometric features, we have found that, with the exception of the aged mice raised in the standard environment, which showed great homogeneity in the morphology of astrocytes, two main morphological phenotypes occupy the molecular layer of the dentate gyrus in adult and aged female albino Swiss mice. We also discovered that relatively few morphological parameters are sufficient to distinguish the morphological changes in astrocytes associated with environment and age in our sample. Because complexity was the morphological feature that best exemplifies such morphological changes, we will discuss it as a basis for the classification of astrocytes, as well as possible functional implications.

### Aging, astrocytes’ morphological complexity and object recognition

Emerging evidence indicates that the number of cells that express biomarkers of cellular senescence increases with aging and astrocytes in the aging brain express characteristics of senescence-associated secretory phenotype. Indeed, aged astrocytes exhibit increased intermediate GFAP- and vimentin-positive filaments, increased expression of several cytokines (TNFα, IL-1β, and IL-6) in the rat brain [67], and increased accumulation of proteotoxic aggregates [68]. Indeed, aged astrocytes exhibit increased intermediate GFAP- and vimentin-positive filaments, increased expression of several cytokines (TNFα, IL-1β, and IL-6) in the rat brain [67], and increased accumulation of proteotoxic aggregates [68]. However, the increase in GFAP-positive filaments in the aging brain is not a consensus. As reported elsewhere, changes in astroglia in ageing and neurodegeneration seem to be highly heterogeneous and region-specific [69] and this include significant differences between white and grey matter astrocytic and microglial activation as ageing progresses [70]. In addition, hippocampal astrocyte cultures from adult and aged rats seem to reproduce changes in glial functionality observed in the aging brain and this seem to include a reduction in GFAP expression that may reflect astroglial degeneration at early stages followed by an increase of GFAP at late stages [71]. Independent of the reasons associated to these contradictory findings an important question that remain to be investigated is how these changes affect astrocytic glutamate exocytosis at the entorhinal-to-dentate granular cells (perforant pathway), because through this mechanism, astrocytes participate in synaptic tuning in circuits involved in cognitive processing and the control of mossy fiber-to-CA3 synaptic input [72]. In addition, even in the absence of neurological disease, a more reactive astrocyte phenotype is expressed during aging as part of an increased and maintained pro-inflammatory profile that may be associated with cognitive dysfunction [73]. A decrease in the ability of aged rats to sustain long-term potentiation in the perforant pathway of the dentate gyrus also appears to be associated with microglial activation [74]. Taking these observations together, it would be reasonable to suggest that the number of astrocytes with senescence-associated secretory phenotype may be increased in animals raised in standard conditions (reducing astrocytic complexity) compared with aged mice housed in enriched conditions [74]. Although this was not the case of the “mean astrocyte” of the molecular layer from aged mice of enriched environment, when we analyzed type I and type II separately, type I astrocytes were significantly more complex than type II in this mice group. In contrast, only one morphological phenotype was found in aged mice raised in standard environment and this was morphologically similar to type II astrocytes.

Object recognition enables the unambiguous distinction between new and familiar objects; see [75, 76] for recent reviews. To cope with memory tasks, the brain must accentuate the differences between old and new experiences before coding occurs [25]. For that purpose, medial and lateral perforant pathways transmit spatial and non-spatial information to the dentate gyrus, which is necessary for recognizing object placement (Where?), identity (What?), and timing (When?). Lateral portions of the entorhinal cortex project to the caudal levels of the dentate gyrus and hippocampus, and medial portions of the entorhinal cortex project to the rostral levels [77, 78]. We have learned from earlier experiments that only 6 mo. adult and aged mice from the enriched environment were able to integrate object recognition into a spatial–temporal context [13]. Mice from standard environments were unable to make the appropriate distinctions. In the spatial memory component of episodic-like memory (Where?), young and aged animals housed in enriched conditions spent significantly more time in the displaced than stationary objects. In the identity and temporal memory components (What? and When?), young and aged animals housed in enriched conditions spent significantly more time in the old than recent objects [13]. Coherently, subsequent analysis of the behavior of aged mice found similar results in tasks that engaged episodic-like memory [79, 80] and working and recognition memories [81]. In the present report using object identity recognition, similar results were found. Indeed, all experimental groups recognized the identity of the objects, only mice from enriched environment (both young and aged) succeeded in the placement task, only young mice both from standard and enriched environments succeeded in the timing task but only young mice from enriched environment distinguished the context where the objects were displaced.

### Enriched environment, neurogenesis, spatial memory improvement and glial cells

Neuronal progenitor cells in the subgranular zone continuously proliferate, migrate into de granular cell layer and differentiate into granule cells [82]. These new neurons, which have been implicated in pattern separation [83], are continually generated in the dentate gyrus in the adult hippocampus [35, 84]. Molecular layer perforant path-associated cells contribute to feed-forward inhibition of these granular cells in the adult dentate gyrus [85] and the integrity of these projections seems to be essential to maintain granular dendritic arbors [86] and spatial learning and memory [87]. Medial and lateral perforant pathways transmit to dentate gyrus, spatial and non-spatial information that would be necessary to learn and recognize object placement (Where?), identity (What?) and timing (When?) [88]. The newborn neurons targeted by perforant pathway, seem to increase significantly in the dentate gyrus of rodents raised in an enriched environment, and this has been associated with spatial memory improvements [89, 90]. In line with these observations our findings demonstrated that spatial memory is influenced by both age and environment and that object recognition memory seems to be resistant to both normal aging and impoverished environment of standard cages

During our previous stereological analysis of the dentate gyrus of aged mice raised in standard environment, we observed hyperplasia of astrocytes in the molecular layer compared with equivalent sections from young adults raised in the same conditions. Interestingly, aged mice from enriched but not from standard exhibited the ability to form integrated memories in the spatial–temporal context [13] However, environment and aging affected the molecular layer of the dentate gyrus in an additive way; thus, we speculated that astrocytosis induced by environmental enrichment might have a different functional role from that induced by aging [13]. Paradoxically, our preliminary morphometric analysis in the molecular layer of the dentate gyrus, using a random but not systematic sampling approach, [45] confirmed earlier descriptions in aged rats [44], that hippocampal astrocytes from aged mice, maintained in an enriched environment, were smaller than those from aged mice maintained in an standard environment. In the present report, we confirmed our preliminary report on the “mean astrocyte” molecular layer. However, hierarchical cluster and discriminant analysis revealed two different morphological phenotypes that had their morphologies distinctly influenced by environment and age.

Although the molecular basis of those changes remains to be investigated, it is important to discuss possible implications associated with the influences of aging and the environment on the morphology of astrocytes in the dentate gyrus.

### Possible physiological implications of an increase in astrocytic complexity

It is interesting to discuss possible connections between the quantitative astrocyte morphological response and the cognitive protection observed after environmental enrichment. In the rodent brain, a single astrocyte is the third element of hundreds of thousands of synapses [91–93]. This ramified complex morphological substrate provides the structural basis for functional interactions with neurons, other glial cell processes, and blood vessels [94]. Studies of neuronal stimulation and astrocyte morphology have taught us that astrocytes react to neuronal stimulation by changing their morphology, and ultrastructural analysis of targeted projections from the stimulated region have demonstrated that neural stimulation causes a significant increase in the astrocytic envelopment of excitatory synapses on dendritic spines [95].

**Fig. 8:**
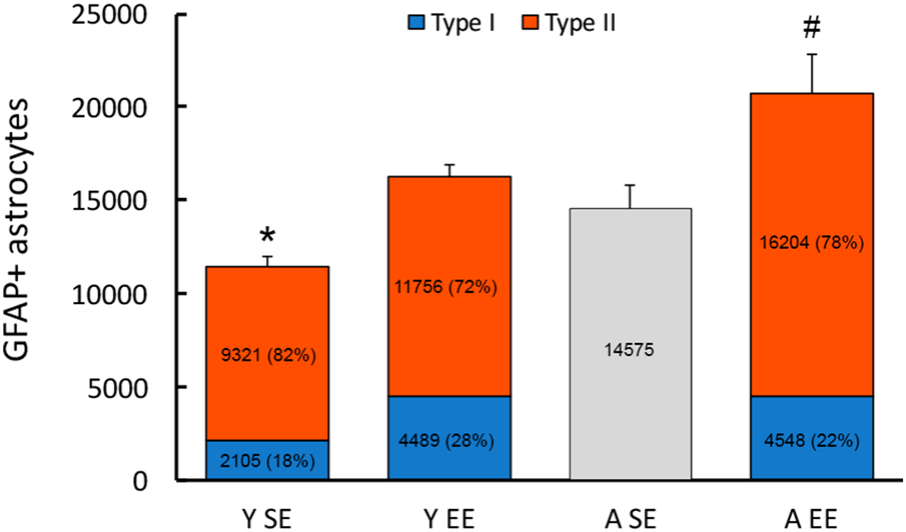
Environment and aging influences on the number and morphology of astrocytes from the molecular layer of dentate gyrus. Relative number of astrocytes morphological phenotypes Type I and Type II as a function of the total number of GFAP immunolabeled astrocytes (GFAP + astrocytes). To estimate these numbers, we used percentage values of type I and type II reconstructed astrocytes in combination with previous stereological data described elsewhere [106]. Note that young mice independent of environment, and aged mice from enriched environment show Type I and II morphological phenotypes, whereas aged mice from standard environment did not. *Y SE* young adult raised in standard environment; *Y EE* young adult raised in enriched environment; *A SE* aged mice raised in standard environment and *A EE* aged mice raised in enriched environment. *SE* standard environment; *EE* enriched environment. (*) and (#) indicate significant differences between the number of total astrocytes from different experimental groups (Y EE vs Y SE; Y EE vs A EE; Y SE vs A SE; A EE vs A SE)

Our findings showed that mice living all life on an impoverished environment lose astrocytic morphological diversity. In contrast, individuals maintained for the same time extent in an enriched environment, did not lose astrocytic diversity. Indeed, 6 months of enriched environment increase 113 % the number of higher complexity astrocytes, and the absolute number of these type I astrocytes are not reduced later in life. aged mice from enriched environment showed the same number of type I astrocytes. Although type II is less influenced by environmental changes (26 % increase in Y EE vs Y SE), it seems that this morphological phenotype is responsible for significant increase in the total number of astrocytes on aged mice from enriched environment (Fig. 8).

Because environmental enrichment is associated with a greater degree of long-term somatosensory/motor and visuospatial stimulation, and in the present report, we observed better performance of an object recognition placement and context tasks in young mice from the enriched environment, with more complex astrocytes, we suggest that at least part of this improvement in hippocampal-dependent tasks performances in young mice, might be associated with astrocytic plasticity. These findings are in line with recent report which demonstrate that housing complexity alters GFAP-immunoreactive astrocyte morphology in the rat dentate gyrus [41]. It is important to highlight that our analysis was done in the molecular layer of dentate gyrus, the main target of the perforant pathway and type I astrocytes were more ramified and longer than type II. Thus, it is reasonable to propose they will affect a higher number of synapses in that layer than type II.

Because type I morphology is affected in higher proportion by aging and environmental impoverishment than type II and aged mice raised in enriched environment had better performances in hippocampal-dependent tasks than aged mice from standard environment it is reasonable to propose different physiological roles for these two phenotypes.

### Hormones and astrocytes

When male Swiss mice were group housed in the laboratory, aggressive interactions between cage mates caused severe injury and stress in the animals. These findings previously described elsewhere, may hamper the validity of experimental results, for review see [96]. To minimize the level of aggression in the cages, female mice were chosen to compose the experimental groups. This choice however, may have included estrogenic changes late in life induced by aging, that may contribute to age-related cognitive impairments and to associated astrocytic morphological changes. Indeed, apart from aging and environment, sexual hormones may change the number [97–99] and morphology [98, 100] of neuroglial cells. In the dentate gyrus, aged C57Bl6J female mice presented 35 % more astrocytes than age-matched males [97] and estrogen and raloxifene changed both the number and morphology of astrocytes of aged female mice [101]. In the present report, aged females may have been depleted of estrogenic protection; thus, we suggest that at least part of the morphological changes detected in aged female mice from both enriched and impoverished environments might be related to estropause. In agreement, ovariectomized female mice given an estrogenic replacement showed significant changes in both the number and morphology of astrocytes in the dentate gyrus compared to an ovariectomized placebo group [101].

Finally, it is important to consider that manipulation-induced stress during behavioral tasks might have altered plasma corticosteroid levels, with implications on astrocytic plasticity [102]. Although we cannot exclude the possibility that different levels of corticosteroids might explain the results the behavioral tests were applied to all animals of all experimental groups which minimizes the possibility that manipulation-induced stress might explain the results.

### Limitations of the experimental design and technical approaches

Comparative analysis of different astrocytes immunomarkers demonstrated that anti-GFAP immunolabeling offers complementary information to anti-S-100ß or anti-glutamine synthetase [103]. Indeed, morphometric analysis of astrocytes, labeled with these three distinct markers revealed region-specific changes in the astroglial morphological phenotypes.

Although the number of GFAP immunolabeled astrocytes may not represent the total number of astrocytes, we and others using GFAP immunolabeling and unbiased stereological methods demonstrated that age [13, 97] and environmental changes [13] were associated with significant changes in the number of the subpopulation of GFAP immunolabeled astrocytes in dentate gyrus. In addition, the environmental enrichment stimulated neurogenenesis and gliogenesis [104], increaseed the GFAP immunolabeled cellular network [105], showing astrocytes with longer branches and higher number of branching points [43, 46]. We [45] and others [32, 44, 46, 103] also demonstrated that as compared to aged mice raised in standard environment, long-life environmental enrichment is associated with shorter GFAP astrocytes branches, lower number of nodes and reduction in the tree surface areas and complexity.

Finally, the influences of environmental enrichment and age on astrocytes’ morphological changes and memory investigated previously in mice and rats using different approaches, models, and techniques [13, 43, 44, 105, 106] were sometimes contradictory. Because different methods, different animal lineages, variations in histological procedures, different stereological protocols, and ambiguities in the definition of the objects and areas of interest were applied, it is reasonable to suppose that at least part of these contradictions might be explained by these differences. To minimize possible sources of variations all samples were obtained with the same tissue processing protocols (perfusion, immunoreaction, dehydration, counterstaining, and clearing) and all data were collected and analyzed with the same unbiased methodology. We also confirmed the results by having different investigators reconstructing the same cells, using the same monoclonal anti-GFAP antibody as a selective marker for astrocytes. Thus, it is expected that non-biological sources were reduced to acceptable levels in the present report [97, 107]. Microscopic, 3-D reconstructions might be affected by non-uniform shrinkage in the z-axis of sections [108]. It was recently demonstrated that sections that the final thickness in the Z-axis is approximately 25 % of the cut thickness after dehydration and clearing [62]. We used this percentage value to implement corrections on all astrocyte reconstructions, assuming 75 % shrinkage of thickness along the z-axis and because the tissue size did not change along X–Y axes after histological dehydration and clearing no corrections were applied to the x/y dimensions.

## Conclusions

Using a combination of stereological sampling approach and three-dimensional reconstruction we found two morphological phenotypes on the molecular layer of dentate gyrus. On average, type I morphological complexity seems to be much more sensitive to age and environmental influences than that of type II. Indeed, aging and environmental impoverishment interact and reduce the morphological complexity of type I astrocytes at a point that they could not be distinguished anymore from type II. Our findings confirm previous reports that the morphological complexity of astrocytes is experience-dependent and suggest at least in young mice that astrocytes of higher complexity may be associated with better performances in object recognition hippocampal-dependent tasks. Although aged mice from enriched environment preserved object recognition, their astrocytes revealed significant degree of shrinkage.

## Additional file

10.1186/s12993-016-0111-2 Object exploration time (s).

## Abbreviations

A EE: aged mice raised in enriched environment
Y EE: young mice raised in enriched environment
A SE: aged mice raised in standard environment
Y SE: young mice raised in standard environment
GFAP: glial fibrillary acid protein
GrDG: granular layer of the dentate gyrus
MolDG: molecular layer of the dentate gyrus
PolDG: polymorphic layer of the dentate gyrus
3-D: three-dimensional
