# Supplementary material for: Age, environment, object recognition and morphological diversity of GFAP-immunolabeled astrocytes
Source: Behav Brain Funct. 2016 Oct 10;12:28. doi: 10.1186/s12993-016-0111-2 (PMC5056502; doi:10.1186/s12993-016-0111-2)
Supplement: Supplementary file 1 — 10.1186/s12993-016-0111-2 Object exploration time (s). [file 12993_2016_111_MOESM1_ESM.docx]

| Table S1. Object Exploration Time (s) | | | | | | | | |
| --- | --- | --- | --- | --- | --- | --- | --- | --- |
|  | Placement | | | | | | | |
|  | Y SE | | Y EE | | A SE | | A EE | |
|  | Stationary | Displaced | Stationary | Displaced | Stationary | Displaced | Stationary | Displaced |
| Mean | 25.45 | 21.88 | 6.117 | 11.63 | 16.51 | 18.3 | 13.21 | 7.711 |
| SE | 3.161 | 3.474 | 1.582 | 2.196 | 3.832 | 5.038 | 3.556 | 3.095 |
|  | Identity | | | | | | | |
|  | Familiar | New | Familiar | New | Familiar | New | Familiar | New |
| Mean | 14.91 | 23.54 | 7.51 | 15.5 | 8.6 | 38.52 | 8.03 | 13.66 |
| SE | 2.569 | 3.664 | 1.467 | 1.73 | 3.042 | 15.3 | 2.332 | 3.754 |
|  | Timing | | | | | | | |
|  | Recent | Old | Recent | Old | Recent | Old | Recent | Old |
| Mean | 19.28 | 10.71 | 26.09 | 7.43 | 35.17 | 10.24 | 30.23 | 22.34 |
| SE | 4.832 | 2.713 | 7.148 | 1.79 | 16.86 | 2.667 | 15.9 | 8.26 |
|  | Context | | | | | | | |
|  | Familiar | New | Familiar | New | Familiar | New | Familiar | New |
| Mean | 19.7 | 22.71 | 9.64 | 24.56 | 21.47 | 12.22 | 16.72 | 14.93 |
| SE | 7.081 | 4.63 | 2.264 | 4.461 | 5.527 | 2.821 | 2.946 | 2.971 |
